# SMAD3 Gene Polymorphisms and Protein Expression as Potential Biomarkers of Susceptibility to Knee Osteoarthritis

**DOI:** 10.7759/cureus.115438

**Published:** 2026-08-30

**Authors:** Amar C Sharma, Dharmendra Kumar, Rajeev Misra, Tridiv Katiyar, Mirza Masroor Ali Beg, Sudeepti R Srivastava, Priti Kumari, Rajeshwar N Srivastava

**Affiliations:** 1 Orthopaedic Surgery, King George's Medical University, Lucknow, IND; 2 Community Medicine and Public Health, King George's Medical University, Lucknow, IND; 3 Molecular Medicine, Sanjay Gandhi Postgraduate Institute of Medical Sciences, Lucknow, IND; 4 Faculty of Medicine, Ala-Too International University, Bishkek, KGZ; 5 Obstetrics and Gynaecology, Health Department, Government of Bihar, Patna, IND

**Keywords:** cartilage, gene polymorphism, knee osteoarthritis, pcr-rflp, rs2289263, rs6494629, smad3

## Abstract

Background

Osteoarthritis (OA) is a progressive degenerative joint disease characterized by cartilage degradation, joint pain, stiffness, and functional disability. Genetic susceptibility plays a significant role in the pathogenesis of OA. SMAD3, an important intracellular mediator of the transforming growth factor-beta (TGF-β) signaling pathway, has been implicated in cartilage homeostasis and in the progression of OA. This study evaluates the association of SMAD3 gene polymorphisms SmaI (C/T; rs6494629) and FokI (A/C; rs2289263) with susceptibility to knee OA and analyzes the protein expression of SMAD3 in serum and cartilage tissue.

Materials and methods

A hospital-based case-control study was conducted involving 200 clinically diagnosed OA patients and 200 healthy controls. Demographic, anthropometric, clinical, and radiological data were recorded. Genomic DNA was isolated from peripheral blood samples. Polymerase chain reaction-restriction fragment length polymorphism (PCR-RFLP) analysis was used for genotyping of SMAD3 polymorphisms. SMAD3 protein expression was analyzed in serum using ELISA and in cartilage tissue using western blotting.

Results

Significant associations were observed between SMAD3 polymorphisms and OA susceptibility. For rs6494629, CT and TT genotypes were significantly associated with increased OA risk compared to the CC genotype (p = 0.009 and p = 0.013, respectively). For rs2289263, CA and CC genotypes showed increased susceptibility to OA (p = 0.02 and p = 0.044, respectively). Serum and tissue SMAD3 expression levels were elevated in OA patients compared to controls.

Conclusion

Altered expression of SMAD3 protein may contribute to OA pathogenesis and could serve as a potential biomarker for disease susceptibility and progression.

## Introduction

Knee osteoarthritis (KOA) is one of the most common chronic degenerative joint disorders affecting elderly individuals worldwide. It is characterized by progressive degradation of articular cartilage, narrowing of joint space, osteophyte formation, subchondral sclerosis, and varying degrees of synovial inflammation. Clinically, OA presents with pain, stiffness, reduced joint mobility, and functional impairment, significantly affecting quality of life. KOA is now considered a disease of the whole joint involving cartilage, synovium, ligaments, and subchondral bone rather than merely articular cartilage degeneration [1,2]. The cause of OA is multi-factorial, including environmental factors, biomechanical factors, metabolic factors, and genetic factors. Age, obesity, joint loading, trauma, and genetic predisposition are some of the major causes leading to the development of the disease. The recent discovery in molecular genetics has led to the discovery of a number of genes that may be associated with susceptibility to KOA [2,3]. The reason for which SMAD3 has garnered considerable interest is that it plays an important role in transforming growth factor-beta (TGF-β) signaling pathways. TGF-β/SMAD signaling pathway is vital in controlling the proliferation, differentiation, extracellular matrix production, and maintaining cartilage homeostasis of chondrocytes. SMAD3 serves as the downstream cellular mediator that relocates into the nucleus once the TGF-β receptors have been activated, controlling the expression of genes responsible for cartilage homeostasis. Disruptions in this signaling pathway can lead to abnormal chondrocyte maturation, extracellular matrix degradation, and progressive cartilage destruction [4,5]. Previous studies have shown that altered SMAD3 expression and mutations in the SMAD3 gene are associated with cartilage degeneration and the progression of OA. Several single nucleotide polymorphisms (SNPs) in the SMAD3 gene have been investigated for their association with OA susceptibility across different ethnic populations. Lu et al. reported a significant association between SMAD3 polymorphisms and increased susceptibility to KOA in the Chinese Han population. Similarly, studies have demonstrated that genetic variations affecting TGF-β signaling contribute to OA pathogenesis and disease severity [6,7]. However, in the Indian population, the SMAD3 gene polymorphism is not much explored; therefore, we hypothesized that SMAD3 polymorphisms (rs6494629 and rs2289263) are associated with increased susceptibility to KOA and that these genetic variants are accompanied by altered SMAD3 protein expression in serum and cartilage tissue.

However, there is limited data on the association between SMAD3 polymorphisms rs6494629 and rs2289263 and KOA in the Indian population. Therefore, the present study was designed to evaluate the association between SMAD3 gene polymorphisms and susceptibility to KOA, as well as to analyze SMAD3 protein expression in serum and cartilage tissue.

## Materials and methods

Subject recruitment

Participants were recruited from outpatient services of the Department of Orthopaedics, King George's Medical University, Lucknow, India, over a four-year period (January 2012 to December 2016) after the calculation of sample size using the formula $\displaystyle N = \frac{Z_{\alpha}^{2}(p \times q)}{d^{2}}$, where the prevalence was 28.7% and power was 80%. Individuals presenting with clinical symptoms indicative of KOA underwent comprehensive clinical evaluation, physical examination, and radiological assessment. The diagnosis of KOA was confirmed using the American College of Rheumatology (ACR) criteria [8] and the Kellgren-Lawrence (KL) radiographic grading system [9]. Patients older than 40 years who met the inclusion criteria and provided written informed consent were consecutively enrolled as cases. The study included 200 patients with clinically diagnosed primary KOA. Concurrently, 200 age- and sex-matched healthy controls, without clinical or radiological evidence of OA and no family history of joint disorders, were recruited from the general population and hospital health check-up clinics. Individuals with rheumatoid arthritis, inflammatory arthritis, autoimmune disorders, congenital bone diseases, metabolic bone disorders, previous major knee trauma or surgery, or those unwilling to participate were excluded. All participants received information about the study objectives and procedures in their local language, and written informed consent was obtained before enrolment. The research work was ethically approved (52 E.C.M. IIA/P3 dated 17th September 2011). Demographic, anthropometric, clinical, and radiological data for all subjects were collected using a structured and pre-validated data collection proforma.

Blood sample collection, DNA isolation and genotyping

Peripheral venous blood samples were collected from all study participants under strict aseptic conditions by trained medical personnel. A total of 5 mL of venous blood was obtained from each participant and divided into two aliquots: 2 mL was transferred into ethylenediaminetetraacetic acid (EDTA)-coated vacutainer tubes for genomic DNA isolation and molecular genetic analysis, and 3 mL was collected into plain sterile vacutainer tubes for serum separation and protein expression analysis. Blood samples in plain vials were allowed to clot at room temperature for 20-30 minutes and then centrifuged at 3000 rpm for 10 minutes. The separated serum was aliquoted into sterile microcentrifuge tubes and stored at -80°C until further biochemical and enzyme-linked immunosorbent assay (ELISA) analyses. EDTA blood samples were temporarily stored at 4°C and subsequently processed for genomic DNA extraction using the standard phenol-chloroform extraction method. Peripheral blood samples underwent red blood cell lysis, followed by isolation of white blood cells. The leukocyte pellet was incubated with cell lysis buffer containing proteinase K to ensure complete digestion of cellular proteins. Equal volumes of phenol, chloroform, and isoamyl alcohol were added to remove proteins and other contaminants. After centrifugation, the aqueous phase containing genomic DNA was separated, and DNA was precipitated using chilled ethanol. The DNA pellet was washed with 70% ethanol, air-dried, and dissolved in TE buffer. Purity and concentration of isolated DNA were assessed spectrophotometrically by measuring absorbance at 260 nm and 280 nm. DNA samples with an A260/A280 ratio between 1.8 and 2.0 were considered suitable for further molecular analysis. Extracted DNA samples were stored at -20°C until genotyping analysis. Genotyping of SMAD3 gene polymorphisms was performed using the polymerase chain reaction-restriction fragment length polymorphism (PCR-RFLP) technique. Positive and negative controls were used, and random samples were selected to cross-check the PCR outcomes regarding the genotypes.

Detection of rs6494629 and rs2289263 polymorphism of SMAD3 gene

rs6494629

A 584-bp PCR product for the SmaI (C/T; rs6494629) polymorphism of the SMAD3 gene was amplified by PCR using the following primers: Forward 5′-ACATTCTGTATCGGAGCCCAGCAT-3′ and Reverse 5′-TTGTTGGTTGCTGGTTTCCTTCGC-3′. The reaction was carried out with 50-200 ng of genomic DNA, 0.2 μmol of each primer, 200 μmol of dNTPs (Fermentas Inc., Hanover, MD, USA), 1.2 mmol of MgCl₂, and 1 unit of Taq polymerase (Fermentas Inc., Hanover, MD, USA) in a total volume of 20 μL. PCR conditions were as follows: initial denaturation at 94°C for five minutes, followed by 35 cycles of denaturation at 94°C for 45 seconds, annealing at 59°C for 45 seconds, and extension at 72°C for one minute, with a final extension at 72°C for 10 minutes (Appendix 1).

rs2289263

A 261-bp PCR product for the FokI (A/C; rs2289263) polymorphism of the SMAD3 gene was amplified by PCR using the following primers: Forward 5′-AAGGAGCAGAGAACACCAGGGAAA-3′ and Reverse 5′-CAAGAGTTGACGACGGGAAGTGA-3′. The reaction was carried out with 50-200 ng of genomic DNA, 0.2 μmol of each primer, 200 μmol of dNTPs (Fermentas Inc., Hanover, MD, USA), 1.2 mmol of MgCl₂, and 1 unit of Taq polymerase (Fermentas Inc., Hanover, MD, USA) in a total volume of 20 μL. PCR conditions were as follows: initial denaturation at 94°C for five minutes, followed by 35 cycles of denaturation at 94°C for 45 seconds, annealing at 58.6°C for 45 seconds, and extension at 72°C for one minute, with a final extension at 72°C for 10 minutes (Appendix 2).

Detection of serum SMAD3 levels

For quantitative determination, serum samples from patients and controls were separated from peripheral venous blood and stored at -80°C until analysis. Circulating SMAD3 levels in serum were measured using commercially available ELISA kits (Human SMAD3 ELISA Kit, Cat# Z01MM-0326-JL488, Creative Biolabs, Shirley, NY, USA), with a minimum detection limit of 0.022 ng/mL. Each sample was assayed in duplicate, and the intra-assay coefficient of variation was less than 10%. The colorimetric reaction was measured at an optical density (OD) of 450 nm using an ELISA reader.

Cartilage tissue sample collection

Cartilage samples were isolated from 15 patients with OA and 10 control patients undergoing orthopedic operations without OA. Articular cartilage samples were taken aseptically from the femoral condyle and tibial plateau sites during the operation. The tissue samples were washed with sterile phosphate-buffered saline (PBS) to remove any blood and tissue contaminants. Cartilage tissues were split into two parts: one was kept in protein stabilization solution for molecular and protein expression studies, and the other part was snap-frozen and stored at -80°C for Western blot analysis. All samples were immediately transferred to the molecular biology laboratory under cold-chain conditions and prepared using a standard procedure for protein isolation and analysis.

Each cartilage sample (cases, n = 15 and controls, n = 10) was considered a separate biological replicate. Protein isolation and immunoblotting were carried out for each biological replicate, with each sample analyzed in duplicate.

Immunoblot Analysis

Protein isolation was done from the tissue using RIPA lysis buffer (HiMedia Laboratories, Mumbai, India) with a combination of both the protease and phosphatase inhibitor cocktails. The concentration of protein was measured using a BCA Protein Assay Kit (HiMedia Laboratories, Mumbai, India), and equal amounts of protein (30-50 µg per lane) were subjected to SDS-PAGE on a 10% polyacrylamide gel and blotted onto polyvinylidene fluoride (PVDF) membranes (Merck Millipore India Pvt. Ltd., Bengaluru, India). The membrane was blocked with 5% non-fat milk in TBST for one hour and incubated overnight at 4°C with primary mouse monoclonal anti-SMAD3 (clone 4C9, Cat. No. WM1201, UCallM Biotech Co., Ltd., Wuxi, China) at a 1:1000 dilution in the blocking solution. This antibody is validated by the manufacturer for Western blot application (suggested dilution range, 1:1000-1:2000), with an observed molecular weight of 52 kDa corresponding to SMAD3 protein. The membranes were then incubated for one hour at room temperature with horseradish peroxidase (HRP)-conjugated goat anti-mouse IgG secondary antibody (dilution, 1:5000; Elabscience Biotechnology Co., Ltd., marketed in India through distributors in New Delhi and Mumbai), and protein bands were detected with an enhanced chemiluminescence (ECL) system. β-actin was used as the loading control to normalize SMAD3 protein expression. Membranes were blotted with mouse monoclonal anti-β-actin antibody (Clone 1B6, Cat. No. E-AB-20092, Elabscience Biotechnology Co., Ltd.; dilution, 1:5000), which detects a 43 kDa band and is tested positive in humans, mice, and rats. The intensity of the band was measured with ImageJ software, and the SMAD3 expression level was normalized to the β-actin band for each biological sample (cases, n = 15 and controls, n = 10), in duplicate. Results showed that the density of SMAD3 protein expression in OA cartilage was significantly higher (21719 ± 2361) than in the controls (15852 ± 2812) (p = 0.006, t-test).

Expression Analysis

SMAD3 levels were measured in human blood serum (cases = 200 and controls = 200) and cartilage tissue (cases = 15 and controls = 10) from recruited subjects. Total protein in serum was quantified using an ELISA following the manufacturer's protocol, while total protein in cartilage tissue was assessed by Western blot.

Statistical Analysis

Statistical analysis was conducted using IBM SPSS Statistics for Windows, Version 23 (Released 2015; IBM Corp., Armonk, NY, USA). Continuous variables are presented as mean ± standard deviation. Group differences were assessed using Student’s t-test for continuous variables and the chi-square test for categorical variables. Odds ratios (ORs) with 95% confidence intervals (CIs) were calculated to assess the association between SMAD3 polymorphisms and OA susceptibility, along with allele frequency by the Hardy-Weinberg equilibrium equation. Statistical significance was defined as a p-value less than 0.05.

## Results

A total of 200 KOA patients and 200 age- and sex-matched healthy controls were included in the study. The demographic and anthropometric characteristics of the study population are summarized in Table 1. No significant differences were observed between cases and controls with respect to age, sex distribution, height, weight, or BMI (all p > 0.05), indicating that the two groups were comparable for subsequent genetic analyses.

**Table 1:** Characteristic of subjects in cases and controls. Values are presented as mean ± standard deviation (SD). Comparisons between cases and controls were performed using the unpaired Student's t-test. Data are expressed with corresponding 95% confidence intervals (CI) for the mean difference between groups. A p-value < 0.05 was considered statistically significant. No statistically significant differences were observed between cases and controls with respect to age, height, weight, or body mass index (BMI), indicating comparability of the two groups at baseline.

Variables	Case	Control	95% CI	p-value
Age	53.43±8.35	53.54±8.30	-1.74 to 1.52	0.89
Height	155.64±8.90	157.12±8.74	-3.21 to 0.25	0.09
Weight	61.97±9.00	62.12±8.81	-1.96 to 1.60	0.86
BMI	26.13±3.34	25.87±4.00	-0.46 to 0.98	0.48

The genotype and allele frequencies of the SMAD3 rs6494629 polymorphism are presented in Table 2. The CT and TT genotypes were significantly more frequent among KOA patients than controls and were associated with an increased risk of OA. Similarly, the combined CT+TT genotype model and the T allele showed significant associations with disease susceptibility.

**Table 2:** Comparison of genotypes of polymorphism in relation to osteoarthritis. Values are presented as frequency (%). Genotype and allele distributions of the rs6494629 (C/T) polymorphism were compared between cases and controls using the chi-square test. Odds ratios (ORs) with 95% confidence intervals (CIs) were calculated using the CC genotype and C allele as the reference categories. A p-value < 0.05 was considered statistically significant. The CT and TT genotypes, as well as the combined CT+TT genotype model were significantly associated with an increased risk of knee osteoarthritis compared with the CC genotype. Similarly, the T allele was significantly more frequent among cases than controls, indicating a positive association between the T allele and susceptibility to knee osteoarthritis.

SNP	Genotypes	Case (n=200) (%)	Control (n=200) (%)	OR 95% CI	p-value
rs6494629 (C/T)	CC	36 (18.0)	60 (30.0)	1 Ref.	
	CT	78 (39.0)	65 (32.5)	2.01 (1.18-3.41)	0.00
	TT	86 (43.0)	75 (37.5)	1.91 (1.14-3.10)	0.01
	CT+TT	164 (82.0)	140 (70.0)	1.95 (1.21-3.12)	0.009
Alleles	CC	150 (37.50)	185 (46.25)	1 Ref.	0.01
	TT	250 (62.50)	215 (53.75)	1.43 (1.08-1.90)	
Allele frequency	CC	0.37	0.64	-	
	TT	0.62	0.53	-	

The distribution of genotypes and alleles for the SMAD3 rs2289263 polymorphism is shown in Table 3. Both CA and CC genotypes were significantly associated with an increased risk of KOA compared with the AA genotype. In addition, the combined CA+CC genotype model and the C allele were significantly associated with OA susceptibility.

**Table 3:** Comparison of genotype of polymorphism in relation to osteoarthritis. Values are presented as frequency (%). Genotype and allele distributions of the rs2289263 (A/C) polymorphism were compared between osteoarthritis cases and controls using the chi-square test. Odds ratios (ORs) with 95% confidence intervals (CIs) were calculated using the AA genotype and A allele as the reference categories. A p-value < 0.05 was considered statistically significant. The CA and CC genotypes were significantly more frequent among cases than controls and were associated with an increased risk of osteoarthritis. Similarly, the dominant genetic model (CA+CC vs. AA) showed a significant association with knee osteoarthritis susceptibility. The C allele was also significantly more prevalent in cases, suggesting that it may confer an increased genetic risk for knee osteoarthritis.

SNP	Genotypes	Case (n=200) (%)	Control (n=200) (%)	OR 95% CI	p-value
rs2289263 (A/C)	AA	62 (31.0)	86 (43.0)	1 Ref.	
	CA	75 (37.5)	61 (30.5)	1.70 (1.06-2.72)	0.02
	CC	63 (31.5)	53 (26.5)	1.64 (1.01-2.69)	0.04
	CA+CC	138 (69.0)	114 (57.0)	1.67 (1.11-2.53)	0.01
Alleles	AA	199 (49.75)	233 (58.25)	1 Ref.	0.01
	CC	201 (50.25)	167 (41.75)	1.40 (1.06-1.86)	
Allele frequency	AA	0.49	0.58	-	
	CC	0.51	0.42	-	

Serum SMAD3 protein levels were further evaluated according to rs6494629 and rs2289263 genotypes. As illustrated in Figure 1A, KOA patients carrying the CT and TT genotypes of rs6494629 exhibited significantly higher serum SMAD3 levels compared with controls. Similarly, Figure 1B demonstrates elevated serum SMAD3 expression among KOA patients carrying the CA and CC genotypes of rs2289263. These findings suggest a possible functional effect of SMAD3 polymorphisms on protein expression.

**Figure 1:**
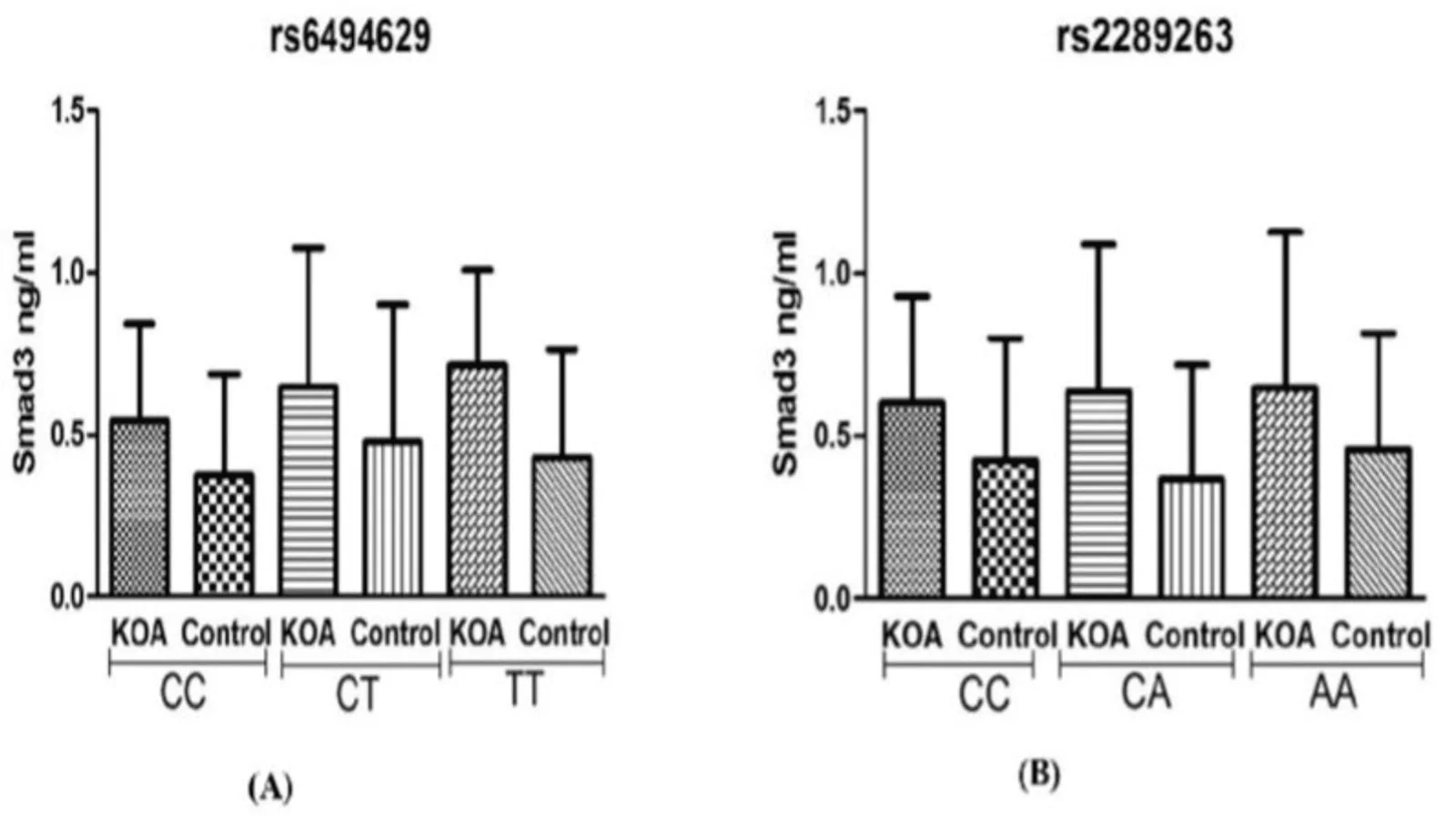
SMAD3 protein expression levels in knee osteoarthritis (KOA) patients and the control group in relation to (A) rs6494629 C/T SMAD3 genotypes and (B) rs2289263 A/C SMAD3 genotypes. (A) rs6494629 C/T SMAD3 genotypes. Comparison of KOA patients and controls: TT genotype, p < 0.0001; CT genotype, p < 0.0006; CC genotype, p < 0.017. (B) rs2289263 A/C SMAD3 genotypes. Comparison of KOA patients and controls: AA genotype, p < 0.006; CA genotype, p < 0.0006; CC genotype, p < 0.004.

To validate the serum findings at the tissue level, SMAD3 protein expression was analyzed in cartilage samples using Western blotting. Representative immunoblots and quantitative densitometric analyses are shown in Figure 2. SMAD3 expression was significantly higher in cartilage tissues obtained from KOA patients compared with controls (p = 0.006), supporting the involvement of SMAD3 in OA pathogenesis. Figure 2A presents representative Western blot images demonstrating increased SMAD3 protein expression in OA cartilage samples compared with controls, with β-actin used as the internal loading control. Figure 2B shows the quantitative densitometric analysis of Western blot bands, revealing a clear increase in SMAD3 protein levels in OA cases relative to controls. Furthermore, Figure 2C summarizes the overall SMAD3 protein expression data, demonstrating significantly higher mean protein levels in OA patients (21719 ± 2361) compared with healthy controls (15852 ± 2812) (p = 0.006). These findings indicate enhanced SMAD3 expression in osteoarthritic cartilage and support a potential role for SMAD3 in OA pathogenesis.

**Figure 2:**
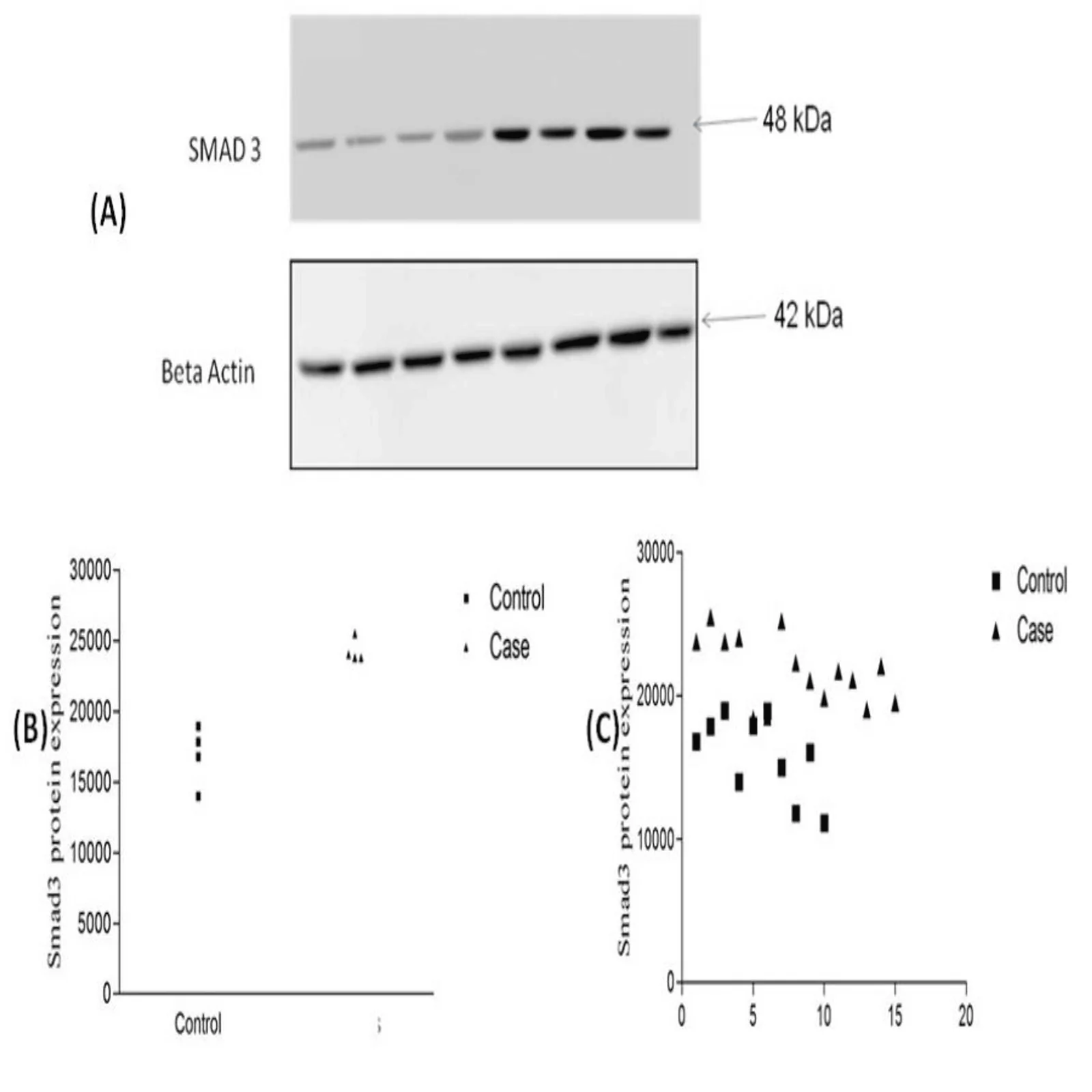
Immunoblot analysis of SMAD3 protein in cartilage tissue. (A) Increased levels of SMAD3 protein are shown, with β-actin used as a protein loading control. (B) Trends show increased SMAD3 protein levels in cases and reduced levels in controls. (C) SMAD3 protein levels were measured in all cases and controls. Data are presented as mean ± SD. SMAD3 protein levels were significantly higher in cases compared to controls (p < 0.006).

Genetic association analysis

The genotype distribution of rs6494629 was compared between OA cases and controls. The CC genotype was observed in 36 (18%) cases and 60 (30%) controls, serving as the reference genotype. The CT genotype was present in 78 (39%) cases and 65 (32.5%) controls, indicating a significantly increased risk of OA (OR = 2.0; 95% CI: 1.18-2.40; p = 0.009). Similarly, the TT genotype was identified in 86 (43%) cases and 75 (37.5%) controls and was also significantly associated with OA susceptibility (OR = 1.91; 95% CI: 1.14-3.10; p = 0.013). These findings suggest that both the CT and TT genotypes of the rs6494629 polymorphism are significantly associated with an increased risk of OA (Table 2).

The genotype distribution of rs2289263 was also compared between OA cases and controls. The AA genotype was observed in 62 (31%) cases and 86 (43%) controls, serving as the reference genotype. The CA genotype was present in 75 (37.5%) cases and 61 (30.5%) controls, showing a statistically significant association with an increased risk of OA (OR = 1.70; 95% CI: 1.06-2.72; p = 0.02). Similarly, the CC genotype was detected in 63 (31.5%) cases and 53 (26.5%) controls and was also significantly associated with OA susceptibility (OR = 1.64; 95% CI: 1.01-2.69; p = 0.044). These findings indicate that both the CA and CC genotypes of the rs2289263 polymorphism may contribute to an increased risk of OA (Table 3).

Detection of serum SMAD3 levels

Serum SMAD3 levels were analyzed according to different genotypes of the rs6494629 and rs2289263 polymorphisms in OA (KOA) cases and controls. For the rs6494629 polymorphism, KOA cases with the CT and TT genotypes exhibited relatively higher serum SMAD3 levels compared to controls, while those with the CC genotype had comparatively lower levels.

For the rs2289263 polymorphism, KOA subjects with the CA genotype showed elevated serum SMAD3 levels compared to controls, while those with the AA genotype had moderate expression levels. Overall, these findings suggest that variations in serum SMAD3 levels may be associated with specific polymorphic genotypes and could contribute to the susceptibility and pathogenesis of OA (Figure 1).

Immunoblot analysis

SMAD3 protein expression was evaluated in OA cases and controls using western blot analysis and quantitative protein expression assessment. Western blot results demonstrated substantially higher SMAD3 protein expression in OA cases compared to healthy controls, whereas the housekeeping protein exhibited consistent expression across all samples. Quantitative analysis indicated that the mean SMAD3 protein level in tissue samples was significantly higher in OA cases (21719 ± 2361) than in controls (15852 ± 2812). The difference was statistically significant (p = 0.006), indicating increased SMAD3 protein expression among OA patients (Figure 2).

Diagnostic significance of serum SMAD3 protein in KOA

To evaluate the diagnostic efficacy of serum SMAD3 protein levels in differentiating KOA cases from normal healthy subjects, ROC curve analysis was performed. The ROC curve for SMAD3 displayed an area under the curve (AUC) of 0.58 (95% CI: 0.53-0.64, p = 0.002), suggesting that the biomarker exhibited limited discrimination between KOA patients and healthy controls. The cut-off point for SMAD3 protein level was found to be 0.24 ng/mL, providing 70% sensitivity and 40% specificity in differentiating KOA from healthy subjects (Table 4 and Figure 3). Even though the sensitivity suggests good capacity for identifying KOA patients, the poor specificity implies that SMAD3 alone cannot provide much diagnostic power as a single confirmatory biomarker due to high false-positive results in normal controls. The above-mentioned results imply that serum SMAD3 levels are moderately correlated with KOA (p = 0.002). Nevertheless, the moderate AUC score and poor specificity indicate that SMAD3 is better suited as an auxiliary diagnostic marker than as a diagnostic test itself. To summarize, this study has shown that serum detection of the SMAD3 protein has diagnostic utility in identifying KOA patients with high sensitivity but poor specificity and, hence, cannot be used alone as a diagnostic test. Therefore, the use of SMAD3 protein in addition to other biomarkers may improve diagnostic efficacy.

**Table 4:** Area under the curve (AUC), sensitivity, specificity, and cutoff value for the prognostic importance of the SMAD3. Receiver operating characteristic (ROC) analysis showing the diagnostic performance of serum SMAD3. The area under the curve (AUC) was 0.58 (95% CI: 0.53-0.64). At the optimal cutoff value of 0.24 ng/mL, SMAD3 demonstrated a sensitivity of 70% and specificity of 40%. The association was statistically significant (p = 0.002).

AUC	95% CI	Sensitivity	Specificity	Cutoff	p-value
0.58	0.53-0.64	70%	40%	0.24 ng/mL	0.002

**Figure 3:**
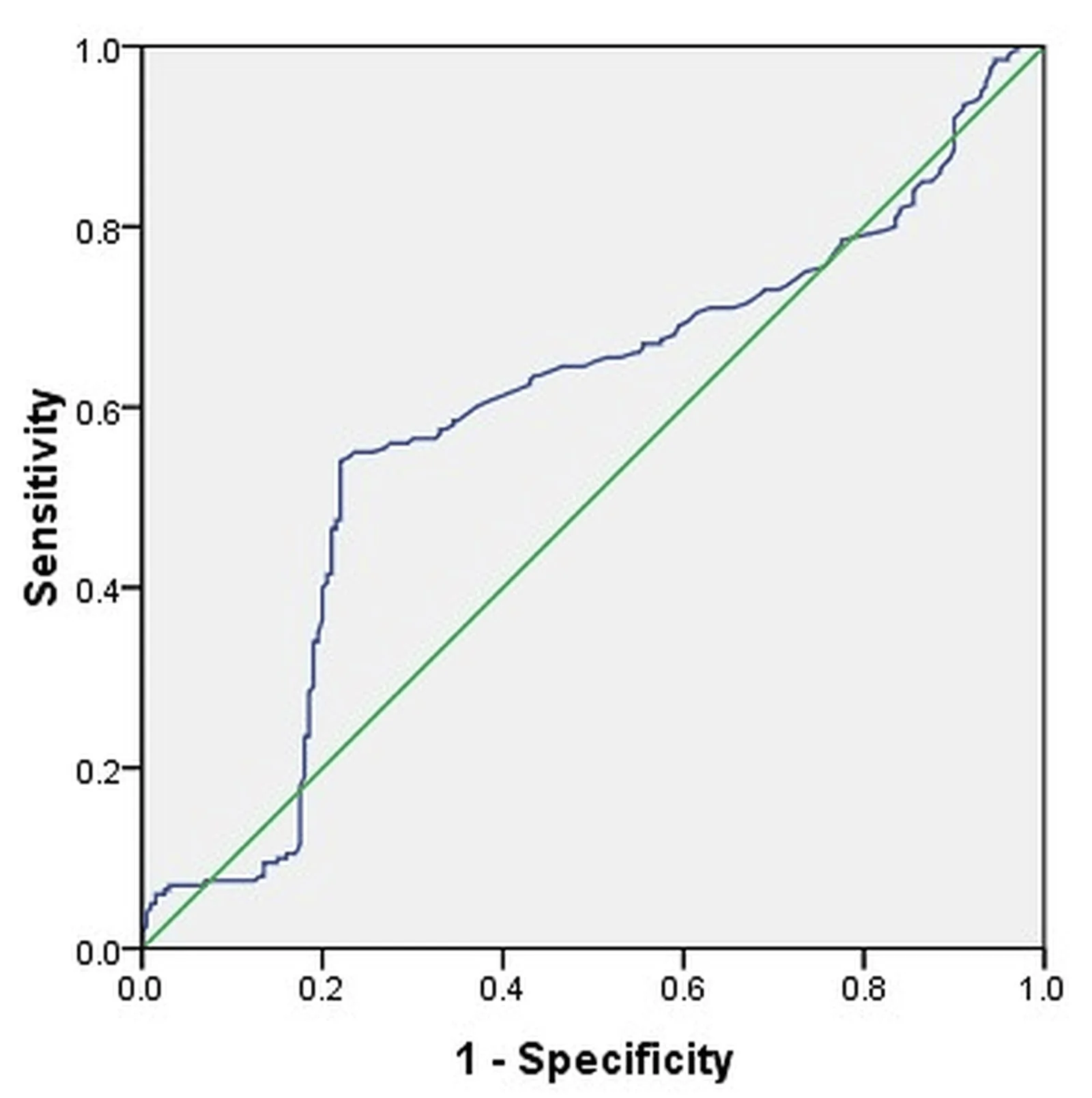
Demonstration of receiver operating characteristic (ROC) curves illustrating the prognostic significance of SMAD3 in knee osteoarthritis. The blue line represents the ROC curve, while the green diagonal line represents the line of no discrimination (AUC = 0.50).

## Discussion

This study investigated the association between SMAD3 gene polymorphisms and SMAD3 protein expression with susceptibility to KOA in the North Indian population. The findings indicate that the rs6494629 and rs2289263 polymorphisms of the SMAD3 gene are significantly associated with an increased risk of OA. Furthermore, SMAD3 protein expression in serum and cartilage tissue was significantly higher in OA patients compared to healthy controls. These results suggest that altered SMAD3 signaling may contribute to OA pathogenesis by disrupting cartilage homeostasis and extracellular matrix remodeling. SMAD3 functions as a key intracellular mediator of TGF-β signaling and is essential for fibrosis, extracellular matrix synthesis, and regulation of chondrocyte differentiation [10]. Disruptions in this signaling pathway can impair cartilage repair and promote joint degeneration. Previous research has shown that mutations and altered expression of SMAD3 are linked to various musculoskeletal and connective tissue disorders [11]. The present study observed a significant association between SMAD3 polymorphisms and OA susceptibility. These findings are supported by Aref-Eshghi et al., who demonstrated increased SMAD3 expression in human osteoarthritic cartilage independent of promoter DNA methylation [12]. Increased SMAD3 expression in OA cartilage may represent a compensatory mechanism in response to chronic cartilage injury and inflammation.

Similarly, Su et al. reported that gene-gene interactions involving TGF-β/SMAD3 signaling pathway polymorphisms significantly influence susceptibility to KOA [13]. Additional experimental evidence supports the involvement of this pathway in OA pathogenesis. Yang et al. demonstrated that inhibition of the TGF-β1/SMAD signaling pathway reduced cartilage injury and slowed OA progression in animal models [14]. Collectively, these findings suggest that dysregulated TGF-β/SMAD signaling directly contributes to cartilage degeneration and inflammatory changes in OA.

Mutation analysis by Yao et al. identified alterations in the SMAD3 gene among patients with OA, providing further evidence for its role in disease susceptibility [15]. Liying et al. reported a significant association between SMAD3 gene polymorphism and OA in a Northeast Chinese population [16]. Meta-analyses by Gao et al. and Hong et al. concluded that SMAD3 polymorphisms significantly increase the risk of OA across diverse populations [17,18]. The present study's findings align with those of Sharma et al., who demonstrated an association between SMAD3 and BMP5 polymorphisms and KOA risk in the Indian population [19]. Zhong et al. observed that genetic variation in SMAD3 increases susceptibility to hip OA in the Chinese Han population [20]. Zhang et al. also reported a significant association between the SMAD3 rs12901499 polymorphism and KOA in Chinese subjects [21]. The TGF-β signaling pathway plays a critical role in maintaining cartilage homeostasis and regulating extracellular matrix turnover. Fang et al. reported that dysregulation of TGF-β1 signaling contributes significantly to OA progression [22]. Similarly, Zhai et al. underscored the importance of TGF-β signal transduction pathways in OA pathogenesis and cartilage remodeling [23]. Recent genetic studies have identified SMAD3 as a novel susceptibility locus for OA. Hackinger et al. demonstrated a shared genetic etiology between OA and bone mineral density, further identifying SMAD3 as a significant OA risk locus [24]. These findings reinforce the biological plausibility of the present study's results. The elevated serum and tissue expression of SMAD3 observed in the current study may indicate persistent activation of TGF-β-mediated inflammatory and fibrotic pathways within osteoarthritic joints. Chronic activation of these signaling mechanisms may promote chondrocyte hypertrophy, fibrosis, osteophyte formation, and cartilage destruction [12-15,20]. Based on our observation, increased SMAD3 expression may not necessarily represent a pathogenic mechanism. Instead, elevated SMAD3 expression may also reflect a compensatory response aimed at maintaining cartilage homeostasis or repairing cartilage injury following chronic mechanical stress and inflammation. Collectively, the findings of this study indicate that SMAD3 gene polymorphisms and altered SMAD3 protein expression play a significant role in OA susceptibility and progression. These results further support the involvement of TGF-β/SMAD signaling in OA pathogenesis and suggest that SMAD3 could serve as an indicator for OA.

Limitations of the study

The study has several limitations. First, it uses a hospital-based design in a North Indian population, which may limit the generalizability of the results to individuals of other ethnic backgrounds. Second, the study does not provide functional validation to determine whether there is a causal relationship between increased SMAD3 expression levels and OA. Third, the lack of a longitudinal design makes it impossible to determine whether increased SMAD3 expression is a consequence or a cause of OA. Finally, the authors did not analyze important environmental and lifestyle factors that may act as confounders, such as physical activity or previous injury.

## Conclusions

This study identifies a potential association between SMAD3 gene polymorphisms rs6494629 (C/T) and rs2289263 (A/C) and increased susceptibility to KOA in a North Indian population. Individuals with variant genotypes (CT/TT for rs6494629 and CA/CC for rs2289263) appear to have a higher risk of developing OA compared to those with wild-type genotypes. Additionally, SMAD3 protein expression was found to be elevated in both serum and cartilage tissues of patients with OA, indicating greater activation of the TGF-β/SMAD signaling pathway during disease progression. These results suggest that SMAD3 genetic variations, along with altered protein expression, contribute to the pathogenesis of KOA. Consequently, SMAD3 polymorphisms and protein expression levels may serve as indicators for identifying individuals at elevated risk and for monitoring disease progression. Further large-scale, multi-center studies and functional analyses are necessary to confirm these findings and to investigate the therapeutic potential of targeting the SMAD3 signaling pathway in OA management.

A significant association was observed between serum SMAD3 protein levels and KOA, with high sensitivity but low specificity, making its use as a standalone diagnostic marker difficult. This biomarker could be used as a complementary marker for screening when combined with other diagnostic techniques or imaging findings. Further studies investigating the use of a panel of markers, including SMAD3, for diagnosing KOA are needed.

## Figures and Tables

**Figure 4:**
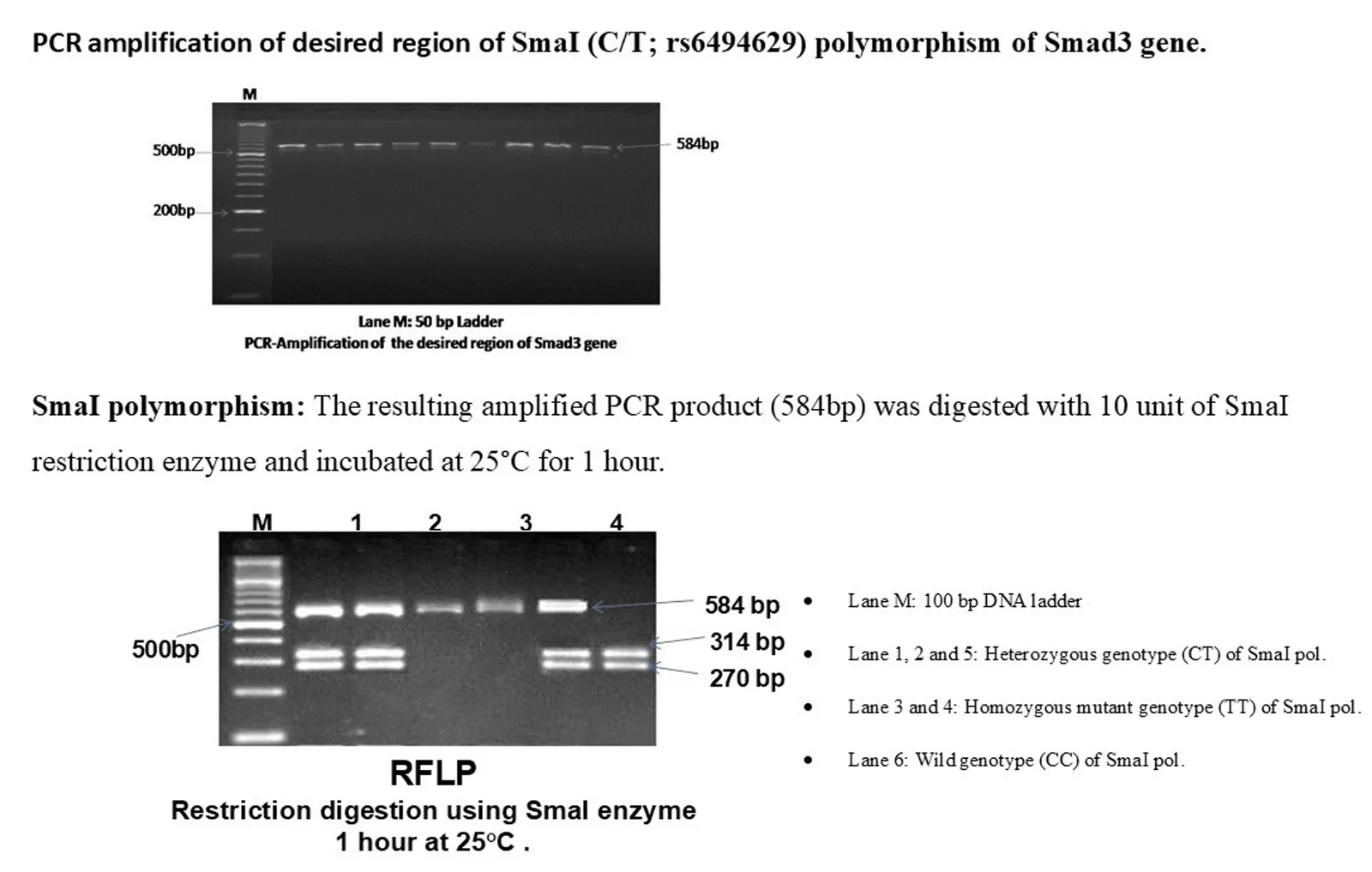
Standardization and optimization of PCR conditions for the SmaI (C/T; rs6494629) polymorphism of the SMAD3 gene.

**Figure 5:**
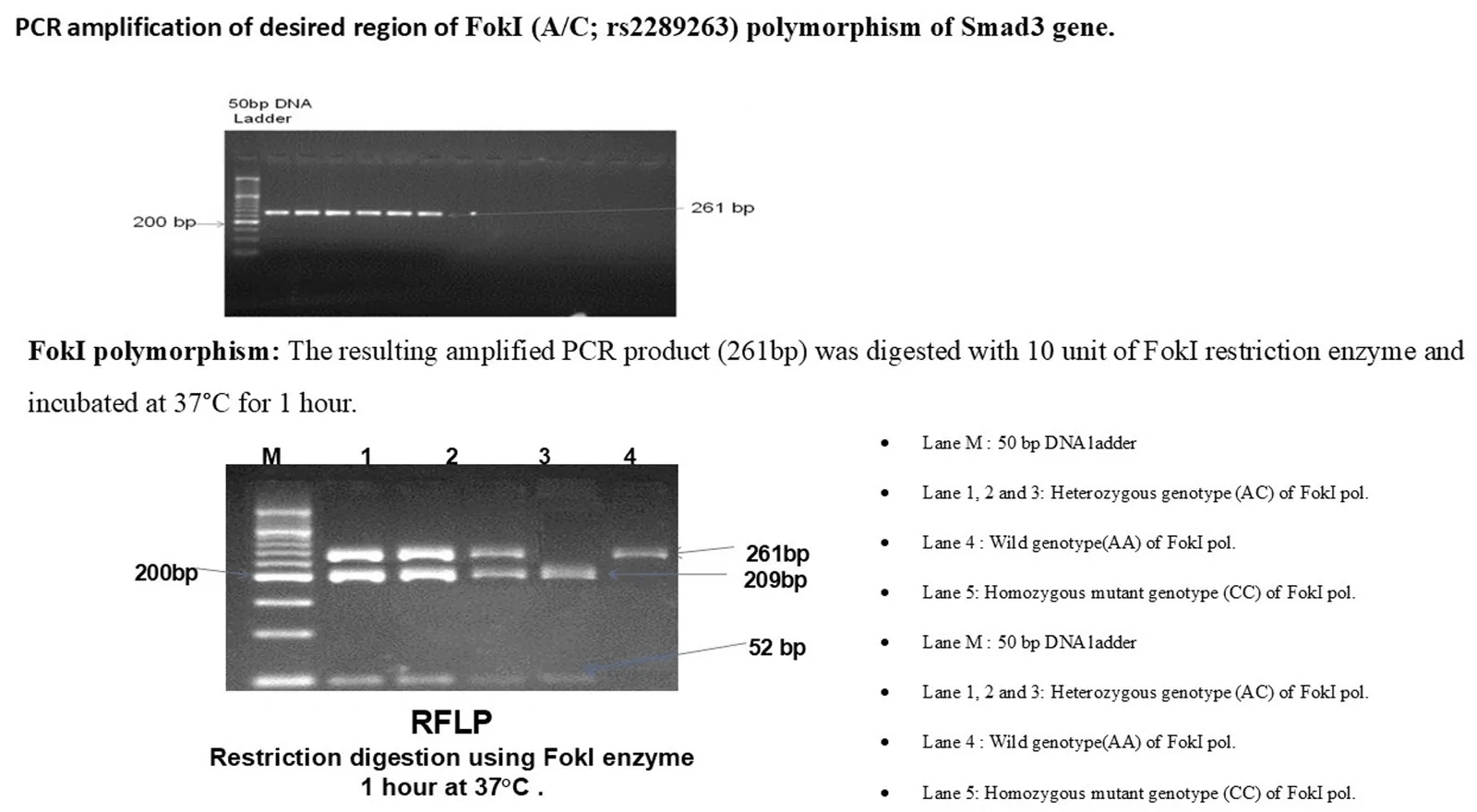
Standardization and optimization of PCR condition for FokI (A/C; rs2289263) polymorphism of SMAD3 gene.

## Appendices

Appendix 1

Appendix 2
